# HOXD9 regulated mitophagy to promote endothelial progenitor cells angiogenesis and deep vein thrombosis recanalization and resolution

**DOI:** 10.1186/s10020-024-00852-5

**Published:** 2024-06-12

**Authors:** Zhang Xiujin, Guo Lili, Fan Jing, Ye Wenhai, Liu Sikai, Shi Wan-yin

**Affiliations:** 1 Department of Radiology, The First Affiliated Hospital of Anhui Medical University, Hefei, 230022 China; 2 Central Laboratory, Shanghai Chest Hospital, Shanghai Jiao Tong University School of Medicine, Shanghai, 200030 China

**Keywords:** Deep vein thrombosis, Endothelial progenitor cells, HOXD9, Mitophagy, Angiogenesis

## Abstract

**Background:**

Deep vein thrombosis (DVT) is a common vascular surgical disease caused by the coagulation of blood in the deep veins, and predominantly occur in the lower limbs. Endothelial progenitor cells (EPCs) are multi-functional stem cells, which are precursors of vascular endothelial cells. EPCs have gradually evolved into a promising treatment strategy for promoting deep vein thrombus dissolution and recanalization through the stimulation of various physical and chemical factors.

**Methods:**

In this study, we utilized a mouse DVT model and performed several experiments including qRT-PCR, Western blot, tube formation, wound healing, Transwell assay, immunofluorescence, flow cytometry analysis, and immunoprecipitation to investigate the role of HOXD9 in the function of EPCs cells. The therapeutic effect of EPCs overexpressing HOXD9 on the DVT model and its mechanism were also explored.

**Results:**

Overexpression of HOXD9 significantly enhanced the angiogenesis and migration abilities of EPCs, while inhibiting cell apoptosis. Additionally, results indicated that HOXD9 specifically targeted the HRD1 promoter region and regulated the downstream PINK1-mediated mitophagy. Interestingly, intravenous injection of EPCs overexpressing HOXD9 into mice promoted thrombus dissolution and recanalization, significantly decreasing venous thrombosis.

**Conclusions:**

The findings of this study reveal that HOXD9 plays a pivotal role in stimulating vascular formation in endothelial progenitor cells, indicating its potential as a therapeutic target for DVT management.

**Graphical Abstract:**

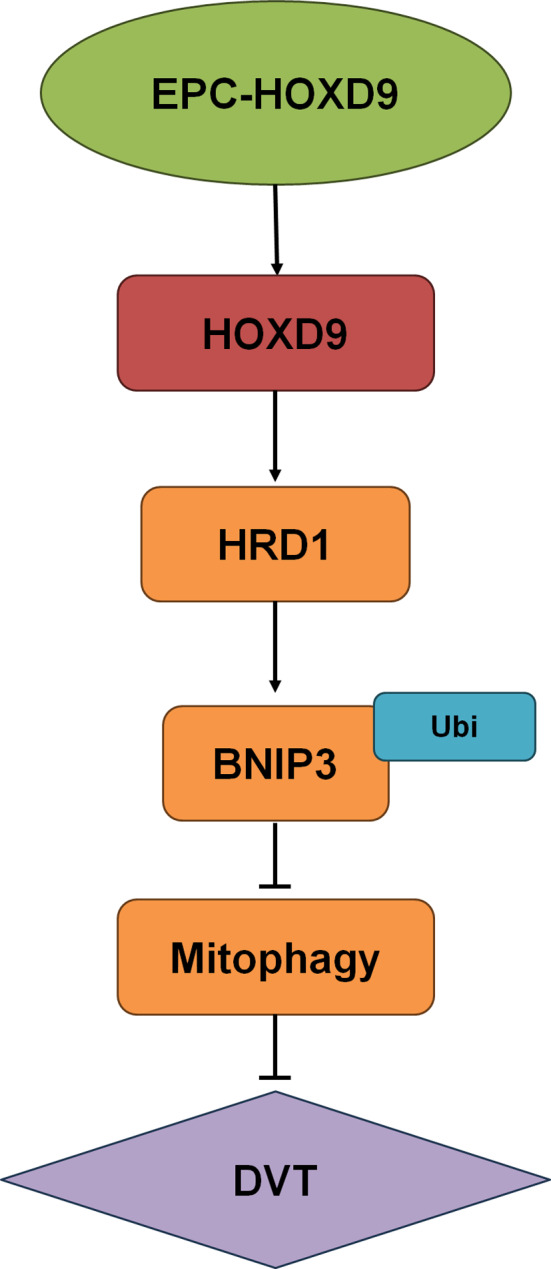

**Supplementary Information:**

The online version contains supplementary material available at 10.1186/s10020-024-00852-5.

## Introduction

Deep vein thrombosis (DVT) is a venous reflux disorder caused by the abnormal clotting of blood in the deep veins, which blocks the lumen and is common in lower limb veins such as the femoral vein (Bokshan et al. 2018; Duffett 2022). Approximately 20–50% of patients develop Post-Thrombotic Syndrome (PTS) after the first DVT occurrence. In severe cases, it can progress to intractable edema, heaviness, hyperpigmentation, pain, and even venous ulcers (Heit et al. 2000). In addition, DVT can be fatal when the thrombus dislodges and progresses to Pulmonary Embolism (PE), leading to sudden heart failure and death (Boon et al. 2018; Tapson and Humbert 2006). Consequently, the quality of life and economic status of DVT patients are severely affected, especially in the presence of PTS (Ueda et al. 2018). Addressing the acute management of DVT and reducing the risk of PTS have emerged as significant challenges for vascular surgeons. Therefore, it is of great clinical importance to further investigate the molecular mechanisms underlying the occurrence and progression of DVT. Equally vital is the exploration of innovative biological therapies, aiming for the identification of precise biomarkers for early detection, alongside discovering targeted prognostic indicators. These efforts hold the potential to revolutionize current diagnostic and therapeutic paradigms for DVT.

Vascular neovascularization represents a pivotal aspect of venous thrombosis development (Santo et al. 2009). EPCs are recruited to the thrombus site, where they expedite thrombolysis and recanalization processes through the secretion of vascular growth factors and cytokines. Their significance extends beyond thrombus resolution, impacting both physiological and pathological vascular neogenesis in adults (Mo et al. 2016). EPCs have emerged as a particularly promising therapeutic avenue in DVT, offering hope for patients whose conditions are refractory to standard treatments. These cells boast the capacity for proliferation, migration, and differentiation into endothelial cells, thereby facilitating neovascularization (Miller-Kasprzak and Jagodzinski 2007). Despite the demonstrated therapeutic potential of EPCs, their clinical application remains hindered by various factors, including smoking, advanced age, diabetes mellitus, cardiovascular risk factors, ischemic disease, and graft vasculopathy. Consequently, devising strategies to enhance EPC recruitment to thrombotic sites and augment their revascularization capabilities is paramount (Salybekov et al. 2022; Xing et al. 2020). . Therefore, such advancements could substantially elevate the role of EPCs in the therapeutic landscape of DVT, offering new horizons for patient care and treatment outcomes.

In this article, we will explore the role of genetically modified endothelial progenitor cells in the treatment of deep vein thrombosis as well as its potential mechanisms.

## Materials and methods

### Culture and characterization of EPCs

The EPCs were isolated from 4–6-week-old male mice as previously described (You et al. 2017). The BM–MNCs collected from various bones were separated and smashed and then separated into layers through density gradient centrifugation (Histopaque 1083, Sigma-Aldrich, USA). Cells cultured in EBM–2 MV medium (Lonza, Basel, Switzerland) supplemented with VEGF, FGF–2, EGF, IGF, etc. Non-adherent cells were removed at 48 h while adherent ones were further incubated until 90% confluence before harvesting using trypsin for passage. DiI–Ac–LDL solution (Beijing Solarbio, China) was added to the medium in the dark for 4 h then fixed with PFA and stained with DAPI observed under the microscope. Flow cytometry analysis was used to identify them through the expression of CD34 (#119,301)/CD31 (#109,502)/VE–cadherin (#138,101)/Flk-1 (#121,902)/CD11b(FITC) (#101,205)/CD45(PE) (#157,603) (BioLegend, USA).

### Immunoprecipitation

Immunoprecipitation was performed as previously described (Yang et al. 2022). 200 µg of total cellular proteins were incubated with antibodies overnight at 4 °C, followed by the addition of 20 µL of protein A/G-agarose beads (sc-2003, Santa Cruz Biotechnology). The precipitates were rinsed four times with lysis buffer, boiled in SDS sample buffer, then immunoblotted with appropriate antibodies.

### Chromatin immunoprecipitation (ChIP)

The EPCs were transfected with mutant or wild-type HDR1 for 24 h. Cells were then rinsed twice with PBS and then cross-linked in a 9 mL culture medium containing 1% formaldehyde at room temperature for 15 min. The reaction was stopped by adding glycine to the final concentration of 125 mM. Chromatin extraction was performed with the High Sensitivity ChIP Kit (ab185913, Abcam) following the manufacturer’s instructions. The chromatin immunoprecipitation with anti-HOXD9 (ab140631, Abcam) antibody was performed with 2 µg chromatin overnight at 4 °C. After cross-link reversal and DNA purification, 1 µL of the eluted DNA was used for qPCR.

### DVT mice model and treatment

Eight-week-old male C57BL/6 mice were obtained from the Shanghai Slake Laboratory Animal Co., Ltd (Shanghai, China). All animal experiments complied with the recommendations of the Animal Care Committee of Anhui Medical University. The protocol was reviewed and approved by the Anhui Medical University Institutional Review Board. As previously reported, IVC stenosis in the DVT model was established (Schonfelder et al. 2017). The HOXD9-EPCs (about 5 × 10^6^) were injected into mice via the tail vein after 24 h of deep venous thrombosis formation. After 7 days, the mice were euthanized, and fresh thrombi were collected. 2 mm sections at the IVC ligation site were fixed with 4% PFA for immunofluorescence and Hematoxylin & Eosin analysis.

### Statistical analysis

The SPSS 21.0 statistical software (IBM Corp. Armonk, USA) was used for statistical analysis. All the data were expressed as mean ± SEM. Analysis was done using Student’s t-test, one-way ANOVA with a Dunnett’s post-hoc test, at *p* < 0.05.

Detailed materials and methods are available in the Supplementary Methods.

## Results

### Characterization of EPCs

**Fig. 1:**
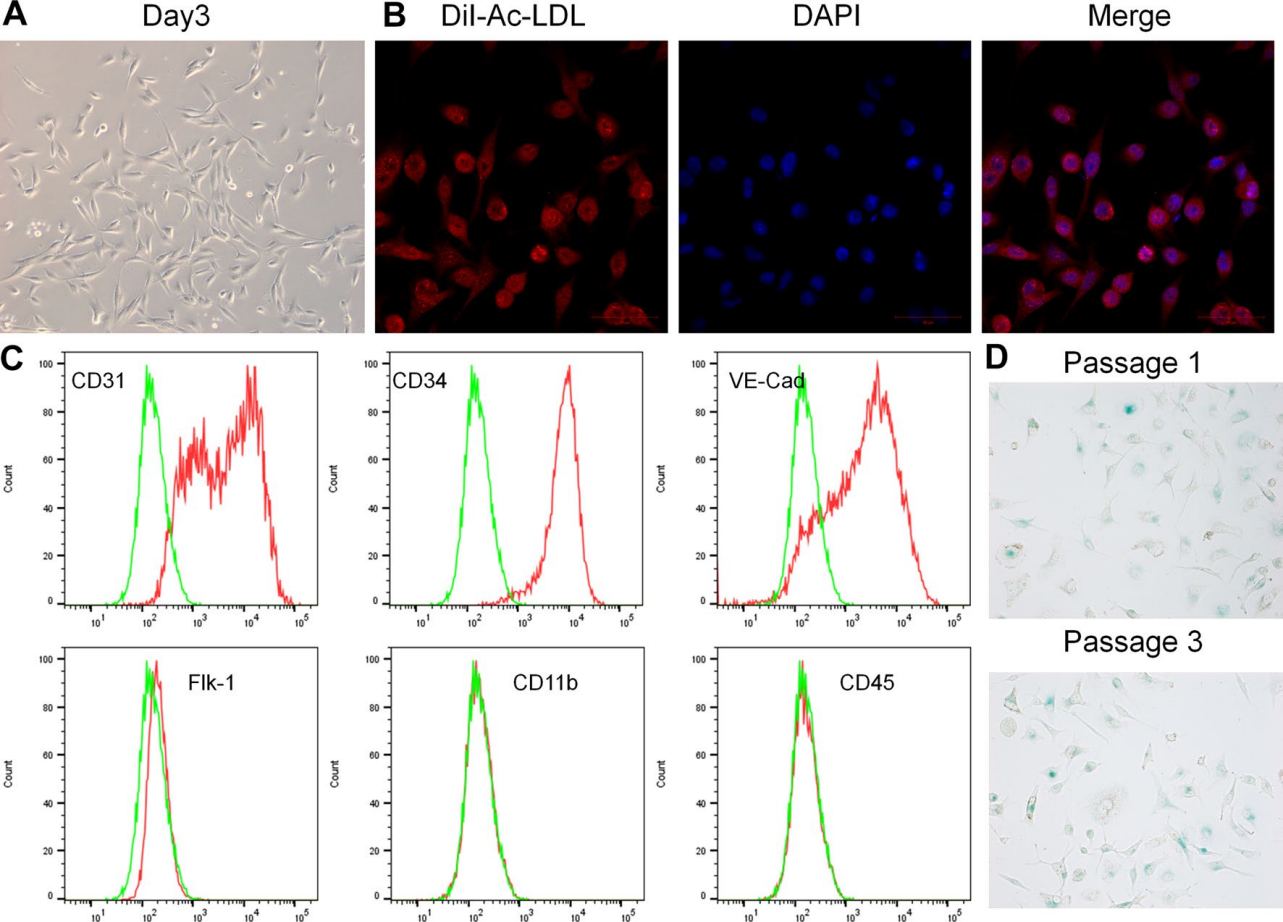
Characterization of EPCs. **A**. Spindle-shaped EPCs were observed on day 3 of BM-MNCs plating. **B**. The image illustrating the EPCs uptake DiI-Ac-LDL (red). The nuclei were stained with DAPI (blue). Scale Bar = 50 μm. **C**. EPC phenotypic analysis by flow cytometry. **D**. Senescent cells were stained with senescence β-galactosidase. Data are presented as the mean ± SD, *n* = 3

EPCs were isolated from the bone marrow of C57BL/6 mice. By day 3, the morphology of adherent cells changed to a typical spindle shape (Fig. 1A). The EPCs were further characterized through the DiI-Ac-LDL uptake assay, and DiI-Ac-LDL uptake by EPCs was confirmed (Fig. 1B). The EPCs were found to express CD34, CD31, and VE-Cadherin, but not Flk-1, CD11b, and CD45. Flow cytometry was performed to identify the surface antigens of EPCs as well (Fig. 1C). Three passages of EPCs were examined for senescence-associated beta-galactosidase activity, which could alter cell functions and paracrine effects. There were no significant differences between passages 1 and 3 EPCs (Fig. 1D).

### HOXD9 enhanced EPCs cell function

**Fig. 2:**
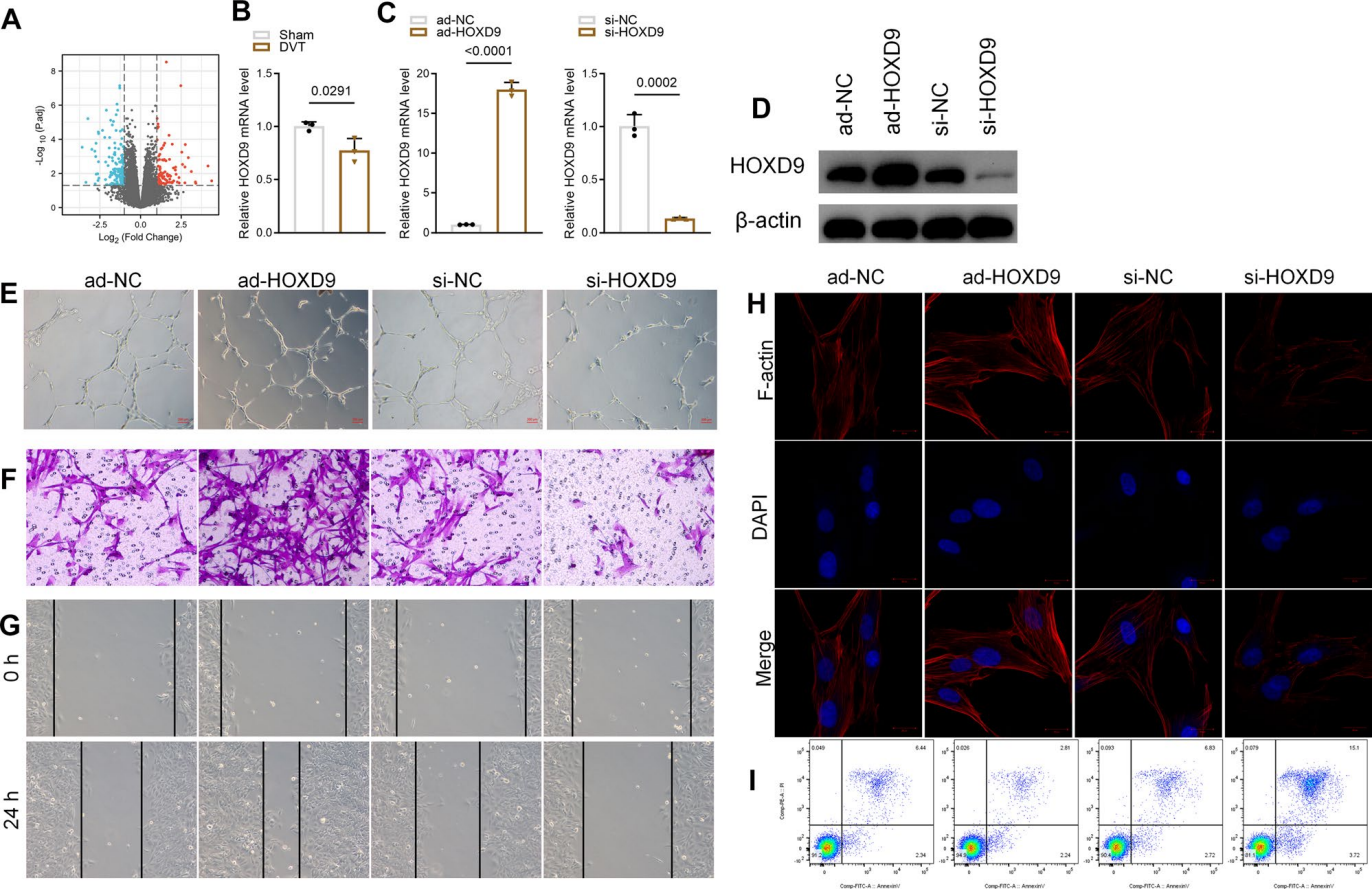
HOXD9 enhanced EPCs cell function. **A**. Volcano plots of differentially expressed genes in GEO datasets (GSE17078). **B**. The mRNA expression of HOXD9 in venous tissue from mice. **C**. The mRNA expression of HOXD3 in EPCs transfected with si-HOXD9, si-NC, ad-HOXD9, or ad-NC for 24 h. **D**. The protein expression of HOXD3 in EPCs transfected with si-HOXD9, si-NC, ad-HOXD9, or ad-NC for 24 h. **E**. Effects of HOXD3 on tube development in EPCs. Scale Bar = 200 μm. **F**. Effects of HOXD9 on cell migration in EPCs. **G**. Wound healing assay of Effects of HOXD9 on cell migration in EPCs. **H**. Effects of HOXD9 on F-actin expression (red) in EPCs. DAPI (blue) was used to stain the nucleus. Scale Bar = 20 μm. **I**. Effects of HOXD3 on apoptosis in EPCs by flow cytometry. Data are presented as the mean ± SD, *n* = 3

We analyzed the differential mRNA of blood endothelial cells in healthy subjects and DVT patients from GSE17078 data by the GEO database (https://www.ncbi.nlm.nih.gov/geo/query/acc.cgi?acc=GSE17078). P-value was analyzed in GEO to correct false-positive results, which < 0.001 and |log2 (fold change)| > 1 was defined as the threshold. The results showed that HOXD9 was significantly reduced in DVT patients (Fig. 2A). We then constructed the mouse DVT model. The mRNA level of HOXD9 in venous tissue of mice is shown in Fig. 2B. The effect of HOXD9 on the function of EPC cells in vitro was then examined. We transfected EPCs for 24 h with small interfering RNA (si-RNA) targeting HOXD9 (si-HOXD9), control si-RNA (si-NC), HOXD9 overexpression adenovirus (ad-HOXD9), or control adenovirus (ad-NC). The ad-HOXD9 significantly increased HOXD9 levels both in mRNA and protein, whereas si-HOXD9 suppressed the level of HOXD9 in EPCs (Fig. 2C and D). EPC tube formation was significantly increased in the ad-HOXD9 group but decreased in the si-HOXD9 group (Fig. 2E). These findings demonstrate that HOXD9 plays a crucial role in EPC-associated angiogenesis. The effect of HOXD9 on EPC migration was evaluated using Transwell invasion and wound healing assays. The Transwell assay revealed that the EPC invasion ability was significantly higher after being transfected with ad-HOXD9, but significantly suppressed in the si-HOXD9 group (Fig. 2F). Furthermore, the wound healing assay yielded similar results, an increase in cell migration capacity with ad-HOXD9 and a decrease in cell migration capacity with si-HOXD9 (Fig. 2G). Additionally, we detected the F-actin expression as it is closely related to cell migration capacity. Immunofluorescence results showed that F-actin filaments were decreased following si-HOXD9, whereas ad-HOXD9 prevented disruption of F-actin filaments (Fig. 2H). Flow cytometry analysis of the role of HOXD9 in EPC apoptosis revealed that si-HOXD9 and ad-HOXD9 enhanced and suppressed EPC apoptosis, respectively (Fig. 2I).

### HOXD9 transcriptionally regulates HRD1 in EPCs

**Fig. 3:**
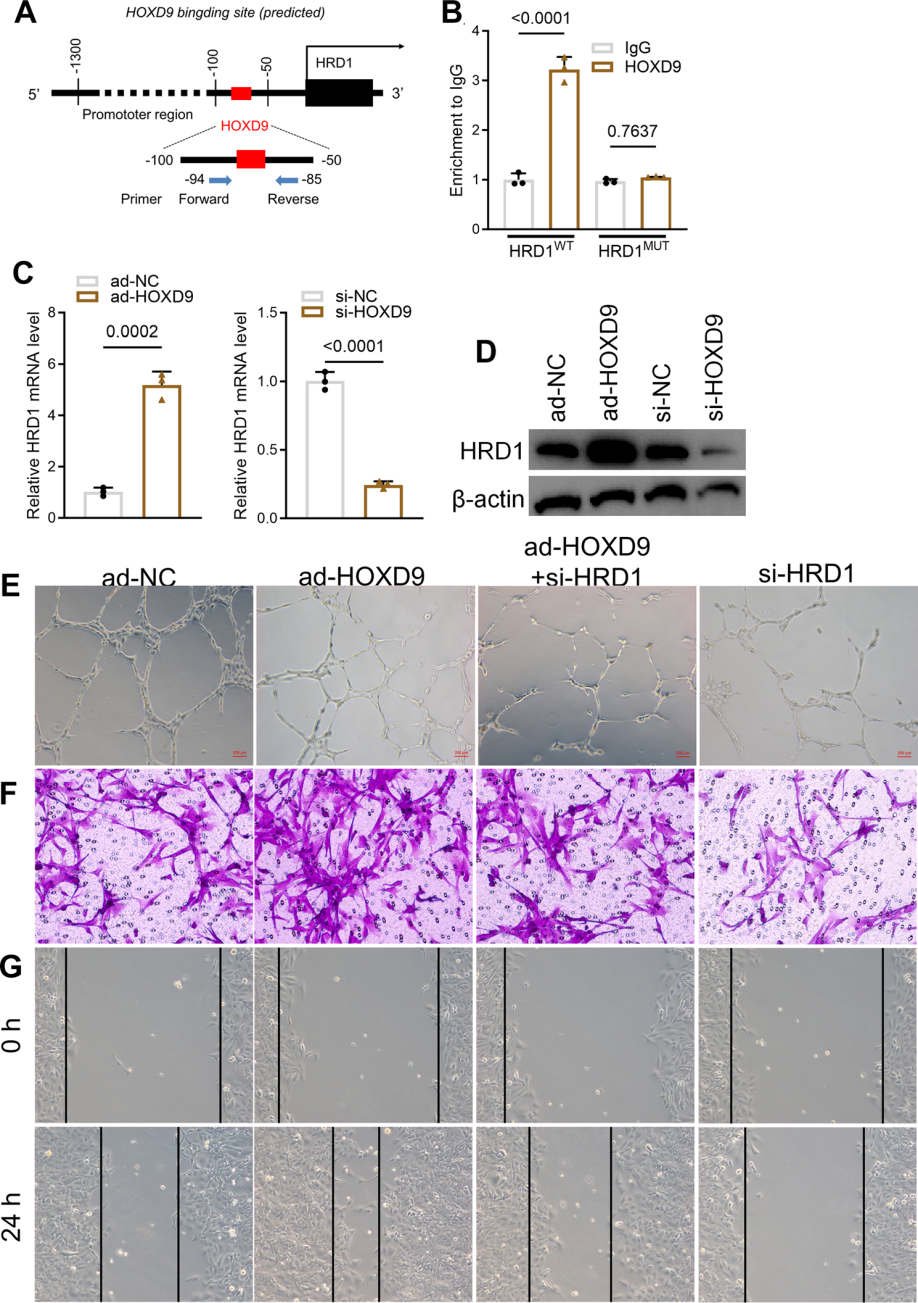
HOXD9 transcriptionally regulates HRD1 in EPCs. **A**. JASPAR database predicts a putative HOXD9 binding site located on the promoter region of mouse *HRD1*. **B**. ChIP assay of the relative enrichment of HOXD9 on the promoter region of mouse *HRD1*. **C**. The mRNA expression of HRD1in EPCs transfected with si-HOXD9, si-NC, ad-HOXD9, or ad-NC for 24 h. **D**. The protein expression of HRD1 in EPCs transfected with si-HOXD9, si-NC, ad-HOXD9, or ad-NC for 24 h. **E**. Effects of HOXD9 and HRD1 on tube formation in EPCs. Scale Bar = 200 μm. **F**. Effects of HOXD9 and HRD1 on cell migration in EPCs as determined by Transwell assay. **G**. Effects of HOXD9 and HRD1 on cell migration in EPCs as evaluated by the wound healing assay. Data are presented as the mean ± SD, *n* = 3

HOXD9 is a transcription factor that has been found to specifically binds to the promoter DNA sequences of downstream genes. Using the JASPAR database (https://jaspar.genereg.net/), we found HRD1 to be a potential binding target for HOXD9 in the promoter region (− 94 to − 85 bp) in EPCs (Fig. 3A). The HRD1 promoter region was then examined using chromatin immunoprecipitation (ChIP). The HRD1 promoter of EPCs was significantly enriched with HOXD9 (Fig. 3B). Moreover, HOXD9 positively regulated the mRNA and protein levels of HRD1 (Fig. 3C and D). We then transfected si-RNA targeting HRD1 (si-HRD1) into EPCs for 24 h. The HDR1 silencing reduced EPC tube formation significantly and reversed the effect of HOXD9 overexpression (Fig. 3E). Furthermore, Transwell migration and wound healing assays showed that si-HRD1 decreased cell migration capacity in EPCs (Fig. 3F and G).

### HOXD9/HRD1 regulated mitochondrial autophagy via PINK1 ubiquitination in EPCs

**Fig. 4:**
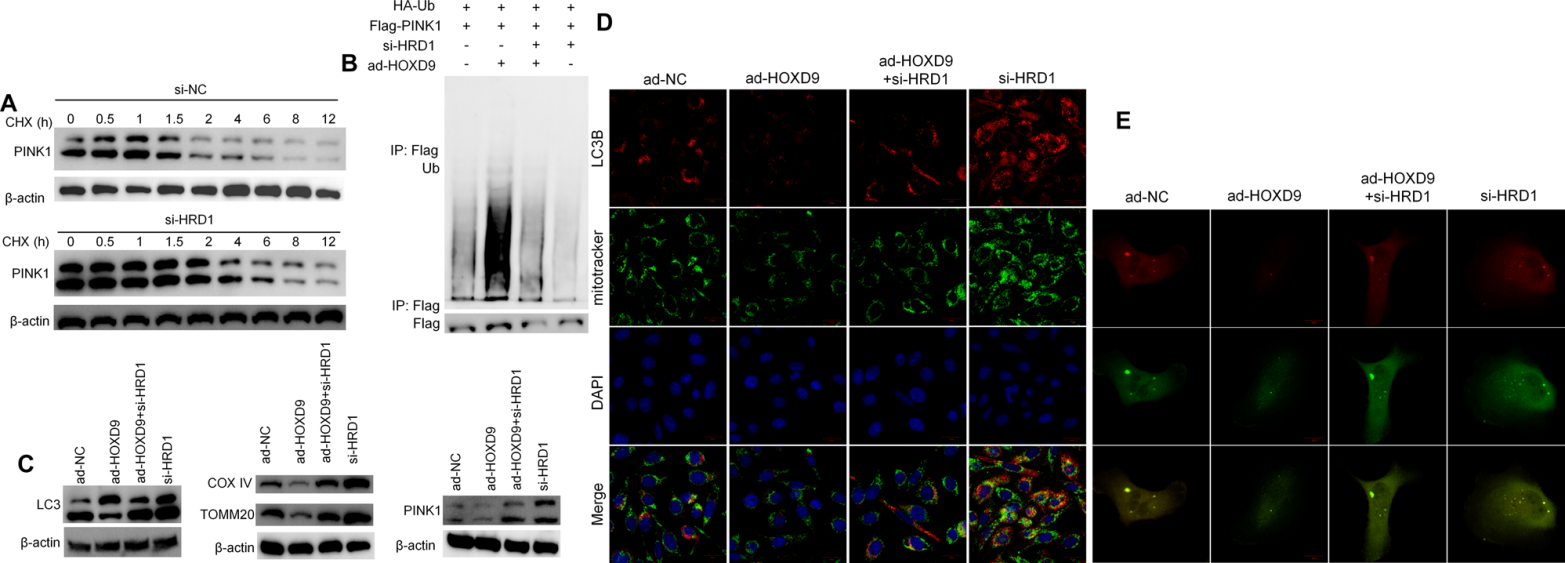
HOXD9/HRD1 regulated mitochondrial autophagy via PINK1 ubiquitination in EPCs. **A**. CHX tracking assay illustrating the effect of HRD1 in EPCs on the change in the half-life of PINK1. **B**. The effect of HRD1 on the ubiquitination of PINK1 in EPCs as determined by Immunoprecipitation assay. **C**. Western blot results showing the protein expression of autophagy-related markers (LC3 and p62), mitochondria-related markers (COX IV and TOMM20), and PINK1 in EPCs. **D**. Effects of HOXD9 and HRD1 on Mitotracker (green) and LC3B (red) in EPCs. The nuclei were stained with DAPI (blue). Scale Bar = 50 μm. **E**. After stably transfected with tandem-labeled mRFP-GFP-LC3, representative images of mRFP-GFP-LC3 vector were shown by immunofluorescent detection. Scale Bar = 20 μm. Data are presented as the mean ± SD, *n* = 3

HRD1 is an E3 ligase that ubiquitinates the line to degrade the mitochondrial autophagy (mitophagy) protein PINK1^15^. Zhang et al. also found that inhibiting EPC cell autophagy restored EPC function (Hassanpour et al. 2018). We used Cycloheximide (CHX) tracking assay to confirm whether the half-life of PINK1 in EPCs changed following HRD1 interference. The half-life of PINK1 increased in EPCs with si-HRD1 compared with the si-NC group (Fig. 4A). This implies that the stability of PINK1 in EPCs decreased after interfered with HRD1. To validate the changes in PINK1 ubiquitination in EPCs, the ubiquitination of PINK1 was identified by immunoprecipitation assay, as shown in Fig. 4B. The ubiquitination of PINK1 was increased in EPCs after transfection with ad-HOXD9 or si-HRD1. Overexpression of HOXD9 enhanced PINK1 ubiquitination, which was reversed in the si-HRD1 group. Western blot analysis revealed that overexpression of HOXD9 increased mitophagy-related proteins such as LC3-B, COX IV, and TOMM20 (Fig. 4C). The Immunofluorescence (IF) analysis demonstrated that mitotracker and LC3B were co-located in EPCs, indicating that the impact of HOXD9 and HRD1 might be associated with mitophagy in EPCs (Fig. 4D). To further confirm this, ad-HOXD9, si-HRD1 and vector cells were infected with an mRFP-GFP-LC3 tandem construct, and the colocalization of mRFP-LC3 and GFP-LC3 puncta was detected. Less puncta were observed in HOXD9 overexpressed cells, indicating HOXD9 overexpression suppressedautophagic flux in EPCs with disrupting the lysosomal function and/or autophagosome-lysosome fusion and si-HRD1 reversed it (Fig. 4E).

### EPC-HOXD9 enhanced thrombus organization and recanalization

**Fig. 5:**
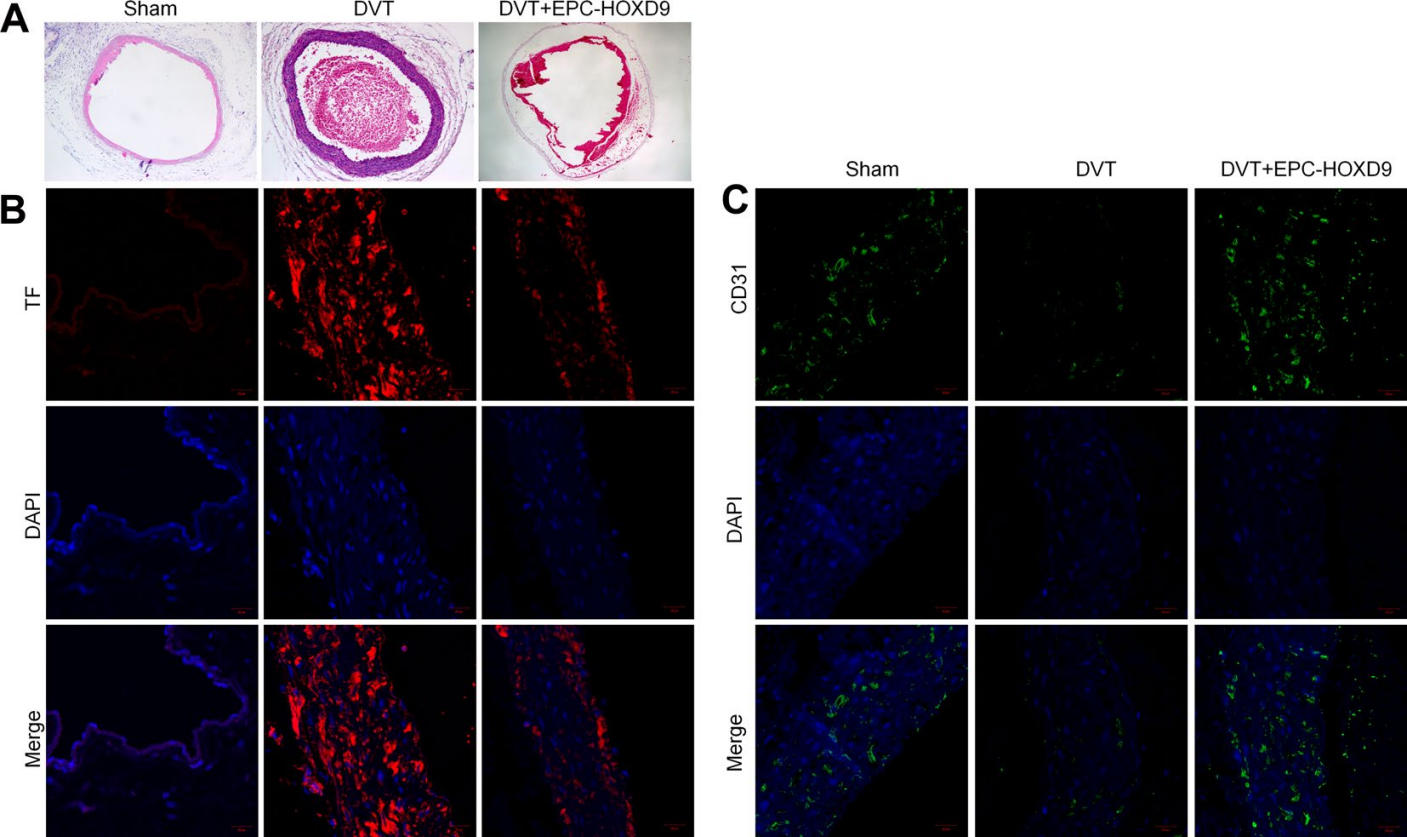
EPC-HOXD9 enhanced thrombus organization and recanalization in vivo. **A**. Representative HE staining images of the thrombus sections. **B**. Representative TF (red) and DAPI (blue) staining images of the thrombus sections. **C**. Representative images of CD31 (green) and DAPI (blue) staining for the thrombus sections. *n* = 3

**Fig. 6:**
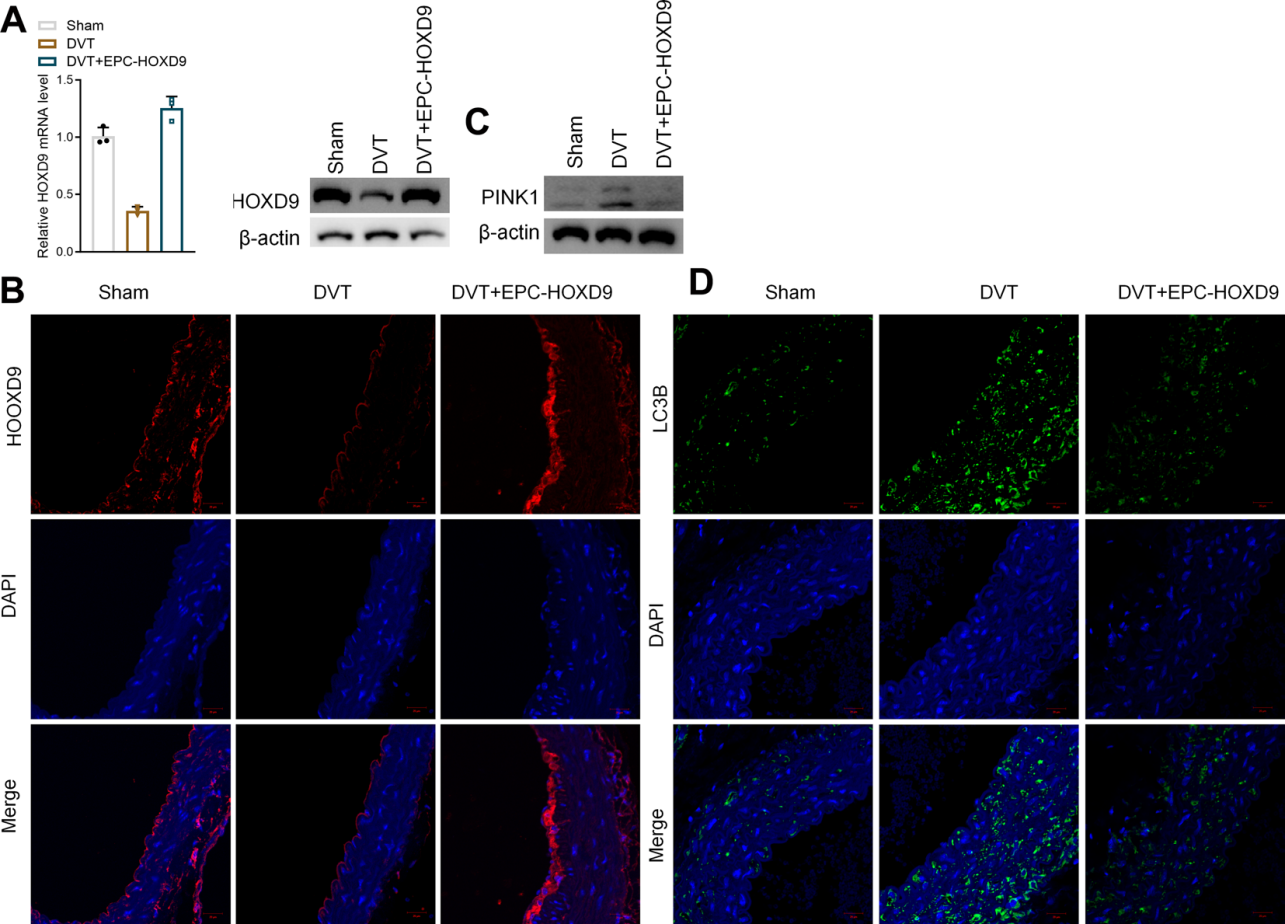
EPC-HOXD9 inhibited mitophagy in vivo. **A**. The mRNA expression of HOXD9 in venous tissue from mice. **B**. Representative images showing HOXD9 (red) and DAPI (blue) staining in the thrombus sections. **C**. The protein expression of autophagy-related markers (LC3 and p62), mitochondria-related markers (COX IV and TOMM20), and PINK1 in mice venous tissue. **D**. Representative images of LC3B (green) and DAPI (blue) staining of the thrombus sections. Data are presented as the mean ± SD, *n* = 3

Tissue and thrombus recanalization play a dynamic and complex role in pathophysiological processes in deep vein thrombosis (Wakefield et al. 1999). We then performed Hematoxylin-Eosin (H&E) and IF staining in mouse models to evaluate the effect of EPC overexpressed HOXD9 (EPC-HOXD9) on thrombus organization and recanalization. Two days after the construction of the mice DVT model, we took the inferior vena cave and stained it with H&E. Thrombus organization in the vessel cavity was typical in the DVT group, with red blood cells stained red and nucleated cells stained blue. In comparison to the EPC-HOXD9 group, there were more nucleated cells and visible channels, as well as smaller thrombi sizes and many newborn vessels (Fig. 5A). The tissue factor (TF) is one of the most potent pro-coagulant factors in the body and the initiator of the coagulation process for physiological hemostasis and pathological thrombosis. IF staining revealed DVT model up-regulates TF protein expression while in the EPC-HOXD9 group, the expression was suppressed (Fig. 5B). CD31, an EC cell-surface marker, was identified to confirm thrombus recanalization. The green fluorescent ring-like structures could be identified as neovascular capillaries encircling the thrombus. The EPC-HOXD9 group had more neovascularization than the DVT group (Fig. 5C). To evaluate the mechanism of HOXD9, we measured the level of HOXD9 in mice venous tissue. The results of RT-PCR and western blot tests revealed that the HOXD9 level was significantly lower in the DVT group. This effect was reversed by exogenous EPC-HOXD9 supplementation (Fig. 6A). Similarly, IF staining confirmed that HOXD9 expression was reduced in the venous tissue of mice in the DVT group, while exogenous supplementation with EPC-HOXD9 increased its expression significantly (Fig. 6B). After supplementation with EPC-HOXD9, we further evaluated the protein levels of autophagy-related markers (LC3 and p62), mitochondria-related markers (COX IV or TOMM20), and mitophagy-related markers (PINK1) in DVT mice. The outcomes of this study revealed that the expression of EPC-HOXD9 led to a decrease in the levels of both autophagy and mitophagy-related markers in the venous tissue of mice. These in vivo findings suggest that EPC-HOXD9 could potentially suppress mitophagy within the venous tissue of mice, thereby ameliorating the symptoms and progression of DVT in these models.

## Discussion

Endothelial progenitor cells are stem cells with high proliferative and multi-differentiation abilities that can differentiate into endothelial cells. They are important in angiogenesis and vascular endothelium repair processes (Asahara et al. 1997; Kalka et al. 2000), and used as biomarkers for some diseases (Miller-Kasprzak and Jagodzinski 2007; Liao et al. 2010). EPCs provide hope for diseases with limited efficacy to conventional clinical therapies, with great application prospects (Santo et al. 2009; Paprocka et al. 2017). However, the number and function of circulating EPCs are limited and easily affected by the internal environment like aging, nicotine, diabetes mellitus, cardiovascular risk factors, ischemic diseases, and transplant vasculopathy. Some cancers may increase the number of circulating EPCs and their biological functions, which may be linked to tumor progression (Paprocka et al. 2017; Naik et al. 2008). These findings suggest that increasing the number and function of circulating EPCs can aid angiogenesis, which could be very important for ischemic or venous occlusive diseases. Therefore, developing strategies to safely and effectively increase the number of EPCs recruited to the thrombus sites can improve the application of EPCs in DVT treatment. Studies have shown that transfection of the VEGF165 gene can significantly increase the angiogenesis and proliferation capacity of EPCs, which may be useful in treating rat venous thrombosis. Studies have also shown that VEGF-mediated EPCs have a strong ability to promote neovascularization in ischemic flap animal models (Yi et al. 2006). Moreover, SDF-1 can increase the angiogenesis ability of EPCs (Liu et al. 2016), indicating that vascular growth factors are beneficial for EPCs proliferation and angiogenesis. However, using these growth factors directly to regulate EPCs may be toxic to other tissues and difficult to control, which contradicts the physiological regulation characteristics of angiogenesis. Furthermore, studies have demonstrated that downregulating miR-103a-3p by targeting PTEN leads to the dysfunction of EPCs in DVT (Zhang et al. 2020), whereas inhibiting miR-195 promotes cell proliferation and vascular formation of EPCs (Mo et al. 2016). Some commonly used drugs for diabetic patients such as metformin, thiazolidinedione class, GLP-1 agonists, DPP4 inhibitors, insulin analogues, and ACE inhibitors can also increase the number and improve the function of EPCs (Desouza 2013). However, due to certain indications and side effects, these drugs are limited in their application scope, and should not be used to treat ischemic diseases or venous reflux disorders. These findings confirmed that HOXD9 promotes endothelial progenitor cell angiogenesis both in vitro and in vivo, promoting thrombus resolution after deep venous thrombosis. In vitro, HOXD9 overexpression enhanced EPC migration angiogenesis, but inhibited cell apoptosis. These findings significantly expand our current understanding of the role of gene-modified EPCs in DVT and elucidate a novel HOXD9 mechanism in EPCs, providing a new target for DVT management. However, in clinical practice, the application of EPCs therapy has certain limitations due to the difficulty of obtaining EPCs and the difficulty of preserving them, which also requires further optimization.

Transcription factors (TFs) are proteins with sequence-dependent affinity to DNA that regulate the transcriptional activity of target genes. The TFs play a key role in vascular endothelial cells in terms of their cellular functions (Donato et al. 2015; Majesky 2018; Ciccarelli, et al. 2011; Ciccarelli, et al. 2008; Poluzzi C, et al. 2014). Transcription factor Homeotic genes (HOX), which belong to the Homeobox family, can recognize specific sequences of other gene promoters, and hence regulate their transcriptional activity (Raines et al. 2015). Several studies have demonstrated the importance of HOX in cardiovascular diseases. Mutations in HOXA11 are the primary cause of genetic defects in platelet generation, structure, and function (Tijssen and Ghevaert 2013). HOXD9 is a member of the HOX family that regulates the development of many diseases. Raines et al. used Hox mutant mice to demonstrate that the HOXD9 gene was crucial in regulating limb development (Raines et al. 2015). Tabuse et al. reported that an elevated HOXD9 expression was involved in cell proliferation and apoptosis in glioblastoma and astrocytoma cells (Tabuse et al. 2011). In cardiovascular research, there are limited reports on HOXD9. Toshner et al. (Toshner et al. 2014) observed that it was highly expressed in human microvascular endothelial cells or human umbilical vein endothelial cells. GEO Data (GSE17078) showed that HOXD9 was significantly decreased in the serum of DVT patients, while our study found that HOXD9 was lowly expressed in the venous tissue of DVY mice. The capacity of capillarization and migration of EPCs was significantly increased after HOXD9 overexpression. To evaluate whether HOXD9 could transcriptionally regulate HRD1, we used the JASPAR database (https://jaspar.genereg.net/) to predict its correlation, confirming that HOXD9 can directly bind to the HRD1 promoter sequence. The ChIP assay further verified these results. Therefore, we concluded a novel mechanism, that HOXD9 enhances the endothelial function of EPCs by promoting the HRD1expression.

A considerable body of research has confirmed that autophagy plays a crucial role in regulating the functions of endothelial cells (Wang et al. 2022). Wortmannin improves blood vessel formation in ketamine-induced cystitis by increasing capillary density and the expression of vascular endothelial growth factor (VEGF) (Lu et al. 2021). Monocyte chemoattractant protein-1 induced protein (MCPIP), through Beclin-1-mediated autophagy, promotes the differentiation of monocytes into endothelial-like cells, facilitating angiogenesis (Niu et al. 2013). Furthermore, the regulation of Beclin-1 by Smad2 transcription is shown to inhibit vascular formation (Pan et al. 2015). Mitochondria are the sites of biological oxidation-phosphorylation, providing Adenosine triphosphate (ATP) to cells via the respiratory chain. They also regulate cell redox potential and signal transduction, apoptosis, and gene expression by producing superoxide anions and other Reactive oxygen species (ROS) (Nunnari and Suomalainen 2012). Lemasters first proposed the concept of mitochondrial autophagy in 2005, which could selectively engulf damaged mitochondria via fusion with lysosomes to maintain their shape and number for the integrity of mitochondrial network function and homeostasis of organismal oxidative system (Lemasters 2005). Inhibition of EPC autophagy has been shown to restore EPC function, whereas an exogenous supply of DVT mice EPCs restore thrombolysis in DVT mice (Palikaras et al. 2017). Our study confirmed that mitochondrial autophagy was activated in DVT mice. After the administration of EPC-HOXD9, PINK1-mediated mitochondrial autophagy was significantly inhibited. In vitro experiments showed that HOXD9 overexpression also suppressed PINK1 expression. The mechanism could be associated with the HRD1 ubiquitination degradation of PINK1. The PINK1/Parkin pathway is the most widely studied non-receptor-dependent mitochondrial autophagy regulatory pathway. PINK1 is a serine/threonine kinase primarily located at the outer membrane of mitochondria, while Parkin is an E3 ubiquitin ligase mainly located in the cytoplasm. PINK1 can recruit Parkin to damaged mitochondria and phosphorylate it to enhance its activity as an E3 ubiquitin ligase, thereby inducing ubiquitination of outer membrane proteins and matrix proteins on mitochondria. Ubiquitin binding protein p62/SQSTM1 accumulates on matrix proteins and binds to LC3 to facilitate the entry of damaged mitochondria into autophagosomes for initiation of mitochondrial autophagy (Palikaras et al. 2017). In this study, we found that HRD1, an E3 ligase located at mitochondria could induce PNK1 ubiquitination degradation and inhibit mitochondrial autophagy of EPCs. However, the study has some limitations including failure to detect the involvement of HOXD9/HRD1 in the regulation of non-receptor dependent pathways such as BNIP3/NIX and FUNDCI, which will be investigated further (Wu et al. 2017; Li et al. 2018).

## Conclusion

We demonstrate that HOXD9 regulates EPCs cell function and DVT structure and recanalization by directly targeting HRD1 and inhibiting PINK1-mediated mitochondrial autophagy. This study adds to the body of knowledge about EPCs therapy for DVT.

### Electronic supplementary material

Below is the link to the electronic supplementary material.

Supplementary Material 1

## Data Availability

The data generated or analyzed during this study are included in this article, or if not, are available upon reasonable request from the corresponding author.

## Abbreviations

DVT: Deep vein thrombosis
EPCs: Endothelial progenitor cells
PTS: Post-Thrombotic Syndrome
si-RNA: Small interfering RNA
ad: Adenovirus
ChIP: Chromatin immunoprecipitation
Mitophagy: Mitochondrial autophagy
H&E: Hematoxylin-Eosin
TFs: Transcription factors
MCPIP: Monocyte chemoattractant protein-1 induced protein
ATP: Adenosine triphosphate
ROS: Reactive oxygen species
