# Supplementary material for: HOXD9 regulated mitophagy to promote endothelial progenitor cells angiogenesis and deep vein thrombosis recanalization and resolution
Source: Mol Med. 2024 Jun 12;30:84. doi: 10.1186/s10020-024-00852-5 (PMC11167931; doi:10.1186/s10020-024-00852-5)
Supplement: Supplementary file 1 — Supplementary Material 1 [file 10020_2024_852_MOESM1_ESM.pptx]

## Slide 1
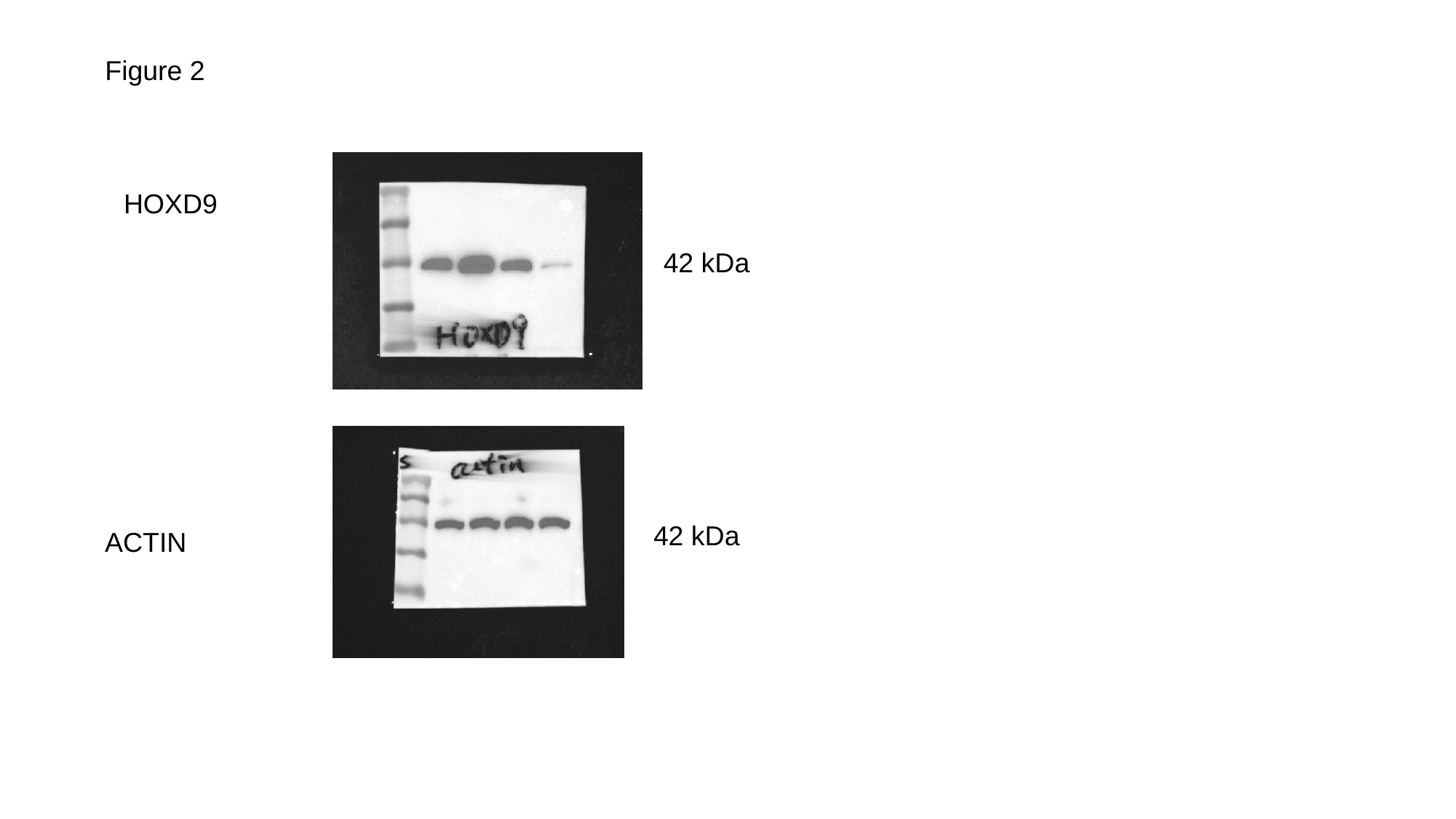

Figure 2
HOXD9
42 kDa
42 kDa
ACTIN

## Slide 2
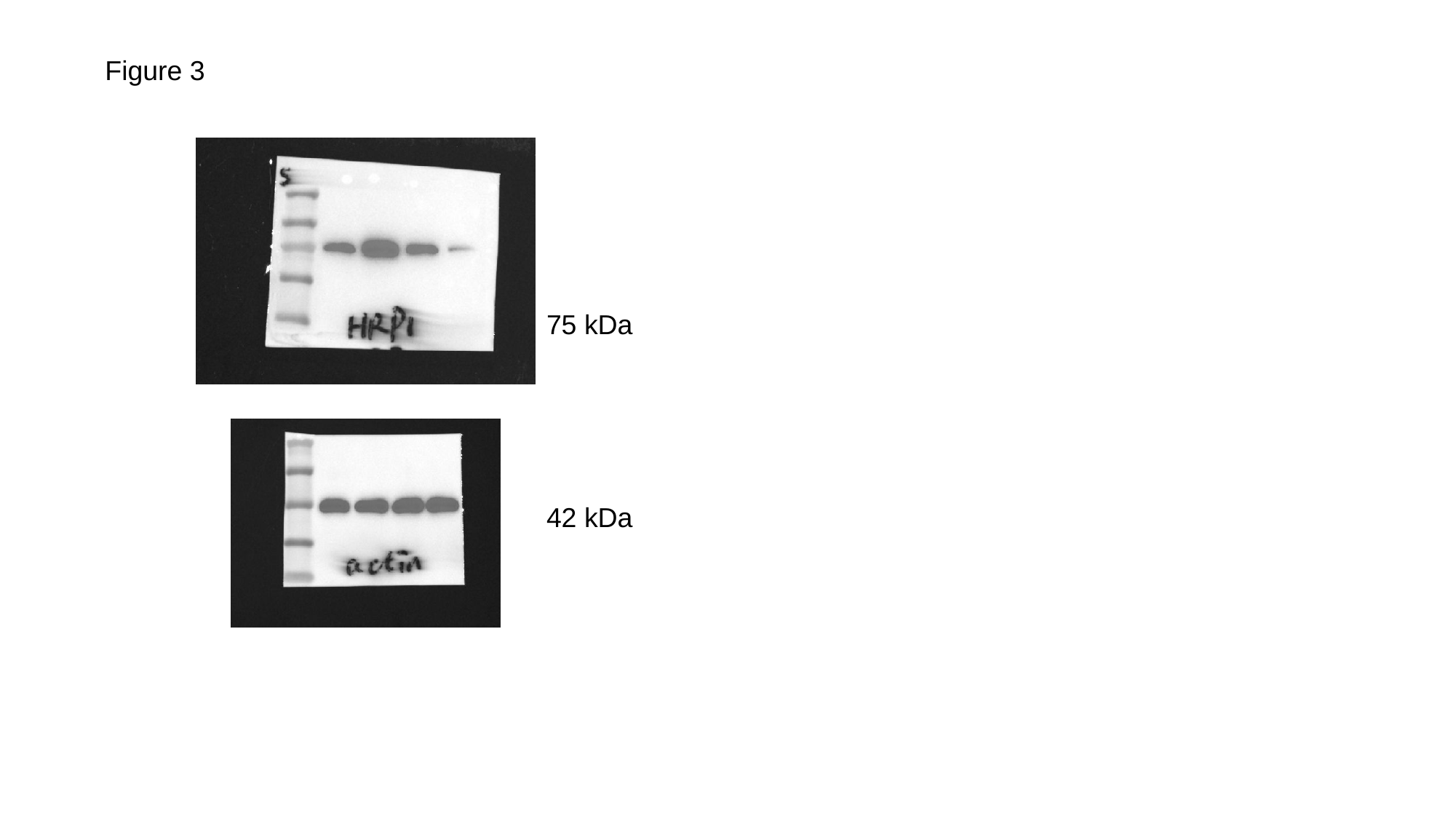

Figure 3
75 kDa
42 kDa

## Slide 3
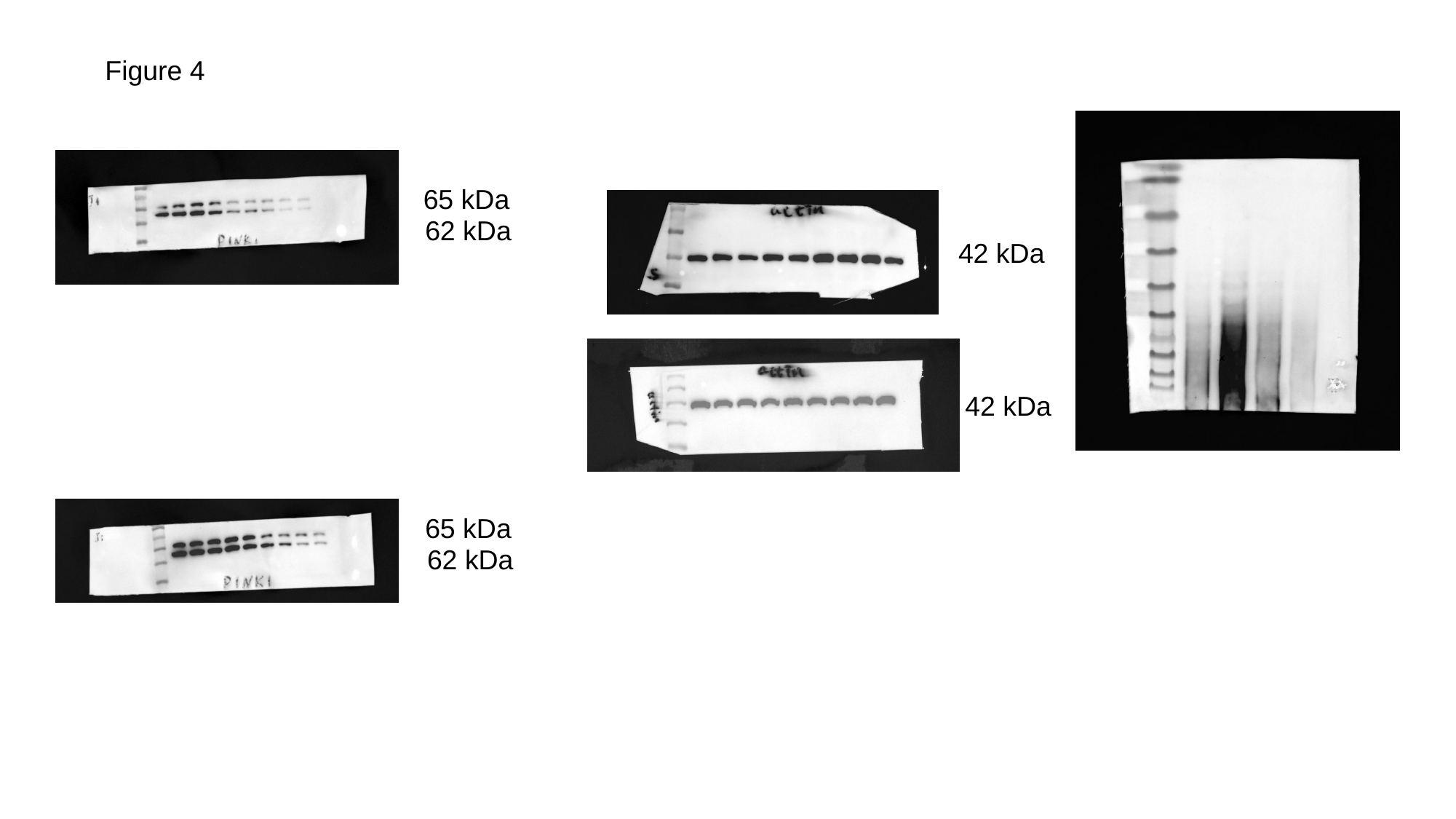

Figure 4
65 kDa
62 kDa
42 kDa
42 kDa
65 kDa
62 kDa

## Slide 4
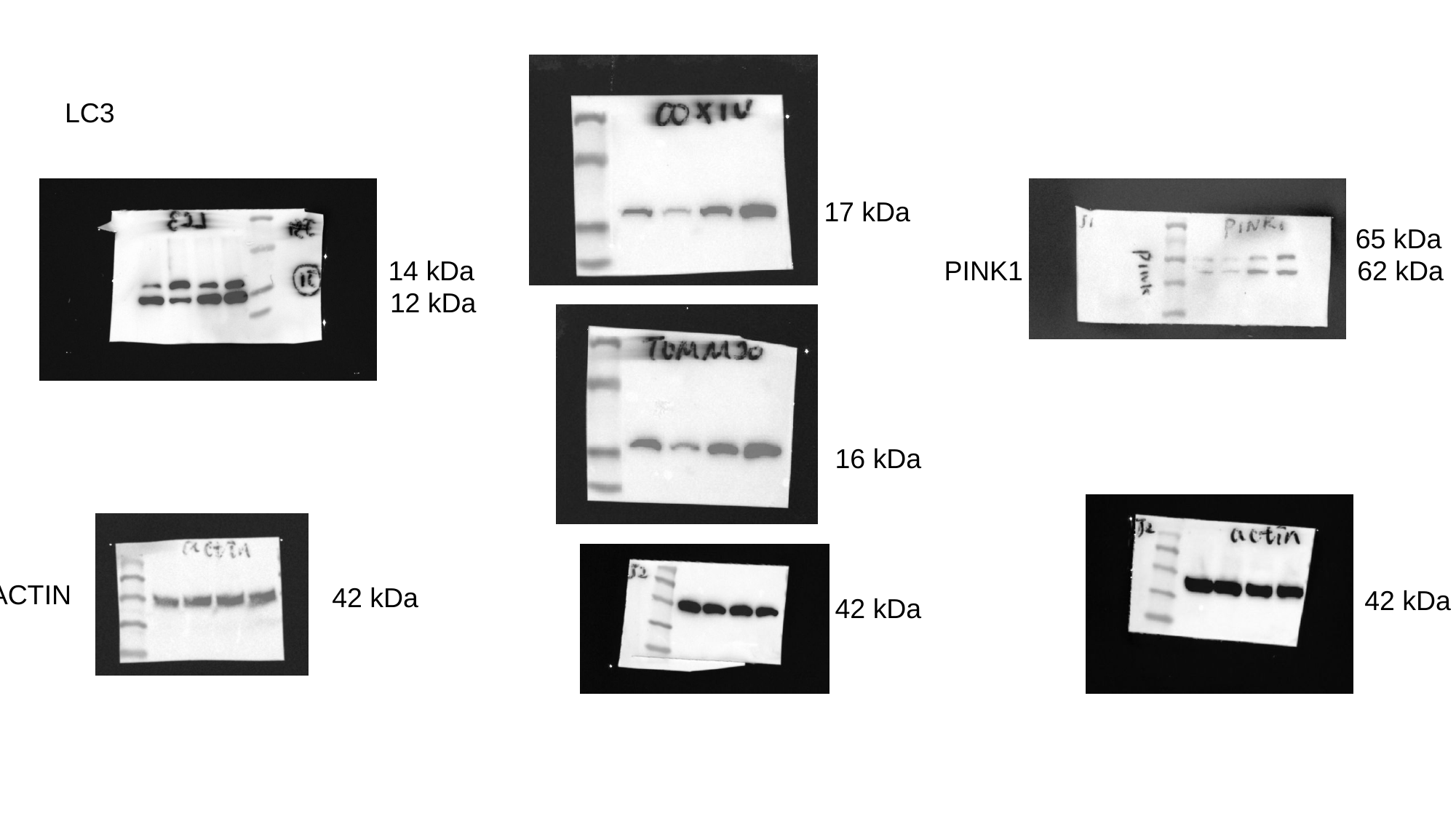

LC3
17 kDa
65 kDa
14 kDa
PINK1
62 kDa
12 kDa
16 kDa
ACTIN
42 kDa
42 kDa
42 kDa

## Slide 5
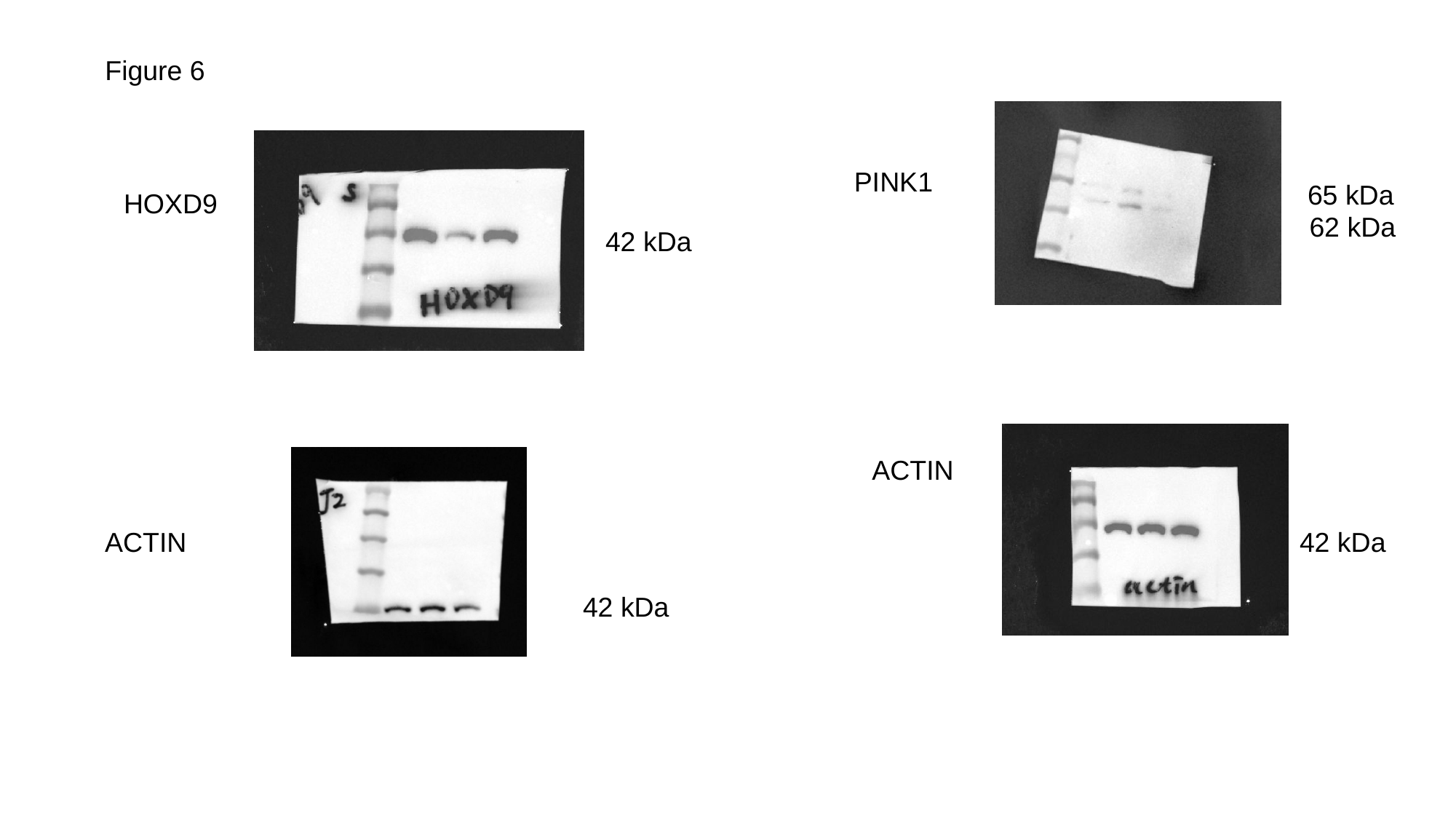

Figure 6
PINK1
65 kDa
HOXD9
62 kDa
42 kDa
ACTIN
ACTIN
42 kDa
42 kDa
